# Supplementary material for: Source apportionment and quantification of liquid and headspace leaks from closed system drug-transfer devices via Selected Ion Flow Tube Mass Spectrometry (SIFT-MS)
Source: PLoS One. 2021 Nov 4;16(11):e0258425. doi: 10.1371/journal.pone.0258425 (PMC8568112; doi:10.1371/journal.pone.0258425)
Supplement: S1 Fig — The chamber was constructed from a desiccator with custom constructed extender section that included glove ports. A battery powered fan was used to circulate the air. (PDF) [file pone.0258425.s001.pdf]

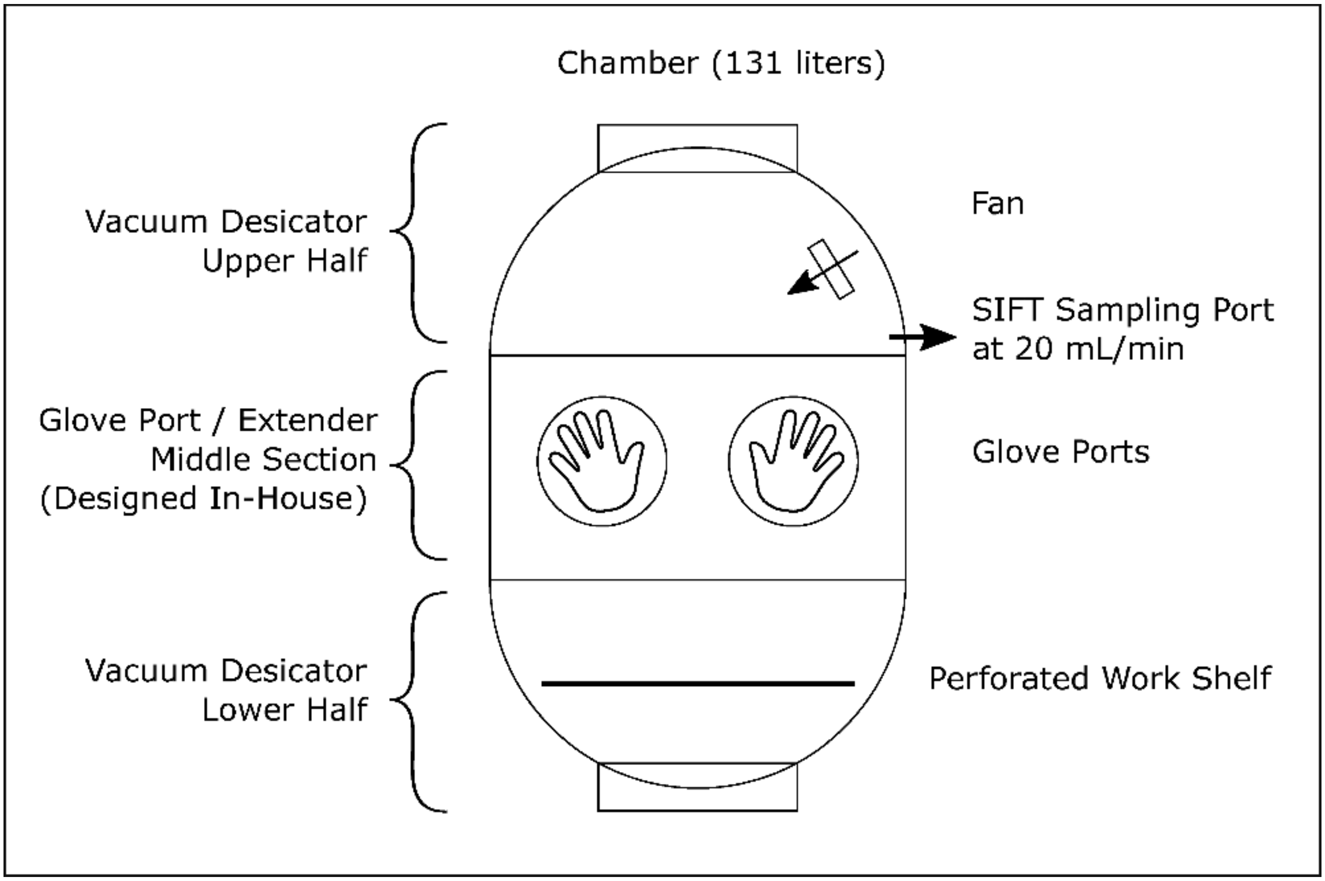


Figure S1. Depiction of the chamber, constructed from the desiccator, with the location of the glove ports, circulation fan, work shelf and SIFT-MS sampling port. The chamber was constructed from a desiccator with custom constructed extender section that included glove ports. A battery powered fan was used to circulate the air.
