# Supplementary material for: Source apportionment and quantification of liquid and headspace leaks from closed system drug-transfer devices via Selected Ion Flow Tube Mass Spectrometry (SIFT-MS)
Source: PLoS One. 2021 Nov 4;16(11):e0258425. doi: 10.1371/journal.pone.0258425 (PMC8568112; doi:10.1371/journal.pone.0258425)
Supplement: S2 Fig — Calibration data plot of the change in acetone vapor response versus liquid volume aliquot of PGAB solution. Calibration curves were made by releasing aliquots of liquid from PGAB solution in increasing volumes, sampled from the test chamber air. (PDF) [file pone.0258425.s002.pdf]

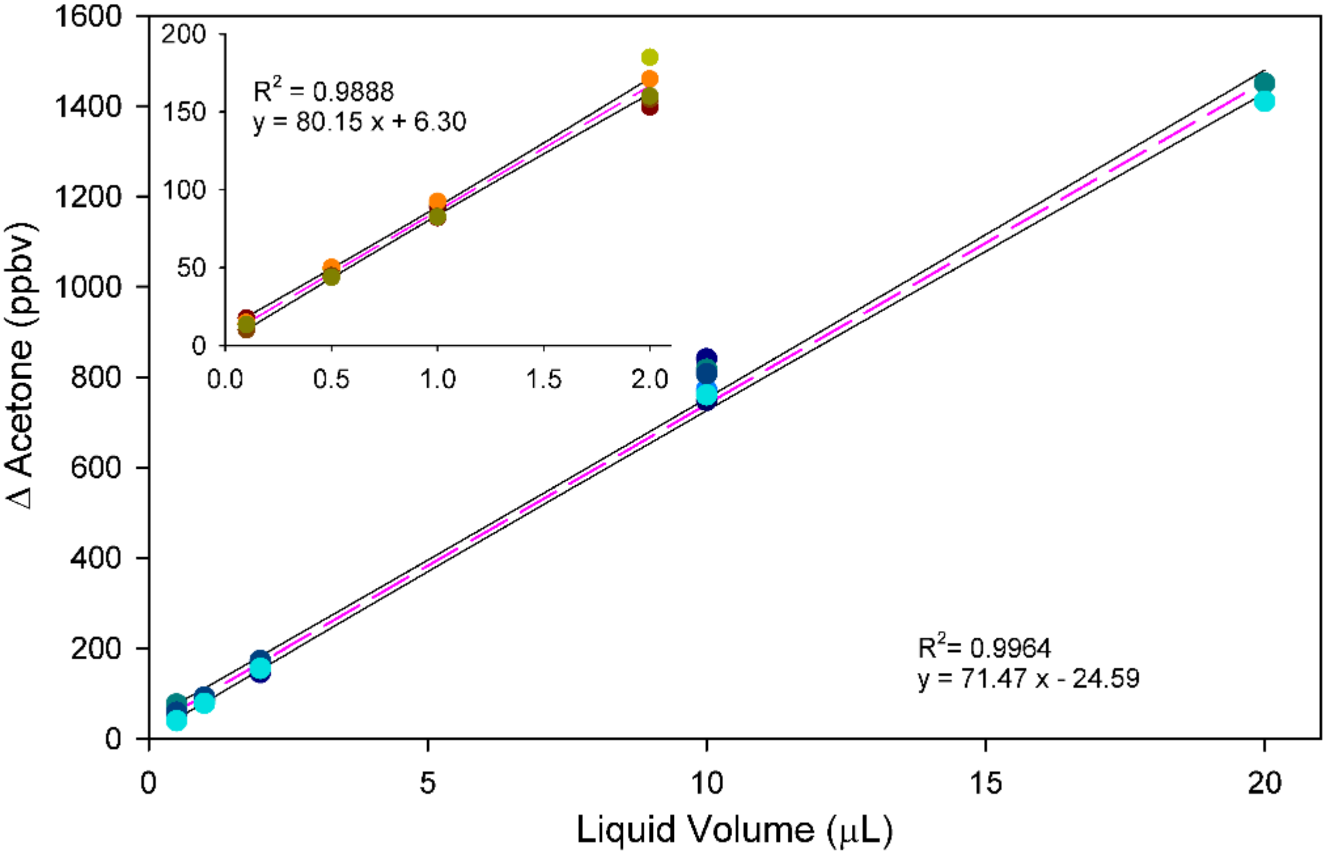

Figure S2. Calibration curves of the liquid PGAB solution. Calibration data plot of the change in acetone vapor response versus liquid volume aliquot of PGAB solution. Calibration curves were made by releasing aliquots of liquid from PGAB solution in increasing volumes, sampled from the test chamber air.
