# Supplementary material for: Source apportionment and quantification of liquid and headspace leaks from closed system drug-transfer devices via Selected Ion Flow Tube Mass Spectrometry (SIFT-MS)
Source: PLoS One. 2021 Nov 4;16(11):e0258425. doi: 10.1371/journal.pone.0258425 (PMC8568112; doi:10.1371/journal.pone.0258425)
Supplement: S3 Fig — Calibration data plot of the change in MTBE response versus headspace aliquot volume from above PGAB solution. Calibration curves were made by releasing aliquots of headspace vapor or liquid from PGAB solution in increasing volumes, sampled from the test chamber air. (PDF) [file pone.0258425.s003.pdf]

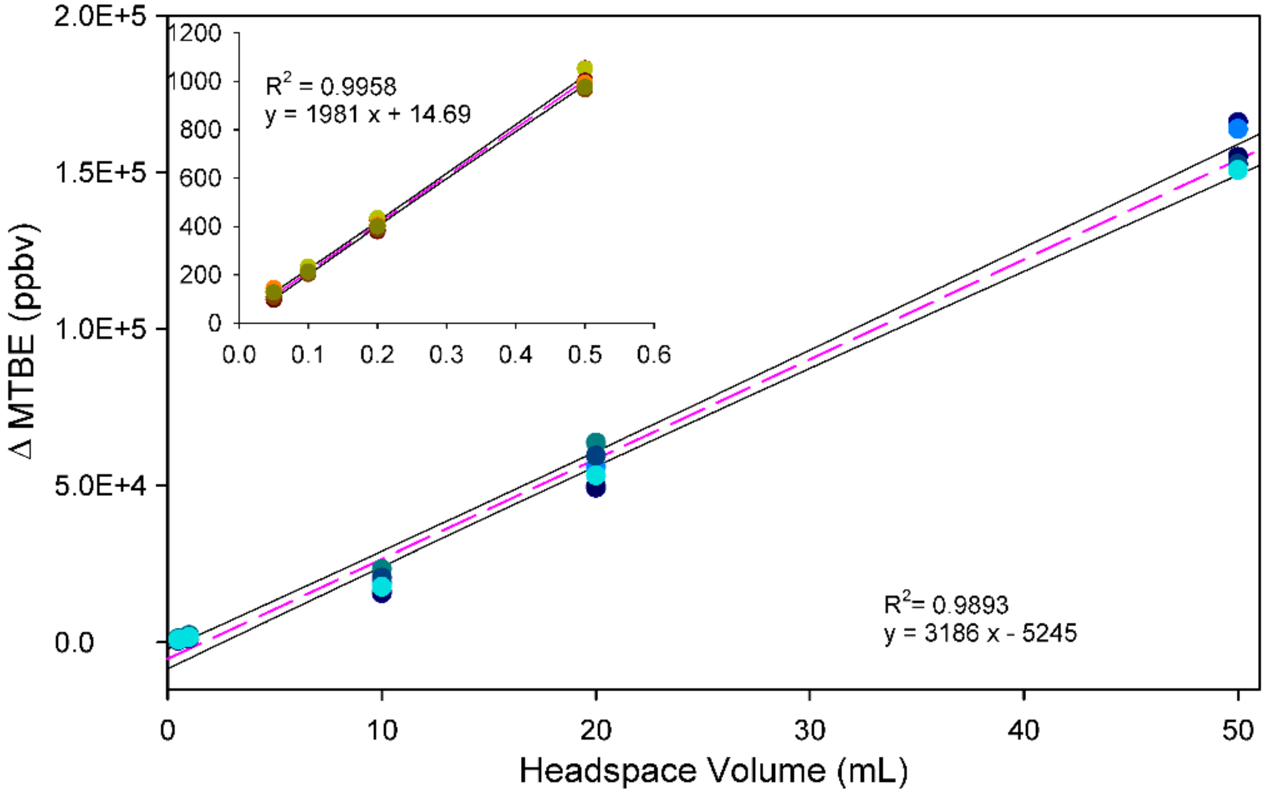

Figure S3. Calibration curves of the headspace of PGAB solution. Calibration data plot of the change in MTBE response versus headspace aliquot volume from above PGAB solution. Calibration curves were made by releasing aliquots of headspace vapor or liquid from PGAB solution in increasing volumes, sampled from the test chamber air.
