# Supplementary material for: Source apportionment and quantification of liquid and headspace leaks from closed system drug-transfer devices via Selected Ion Flow Tube Mass Spectrometry (SIFT-MS)
Source: PLoS One. 2021 Nov 4;16(11):e0258425. doi: 10.1371/journal.pone.0258425 (PMC8568112; doi:10.1371/journal.pone.0258425)
Supplement: S5 Fig — The resulting leak volume of liquid (VL) and headspace (VH) for a given ratio of A/B are shown on the vertical axes. (PDF) [file pone.0258425.s005.pdf]

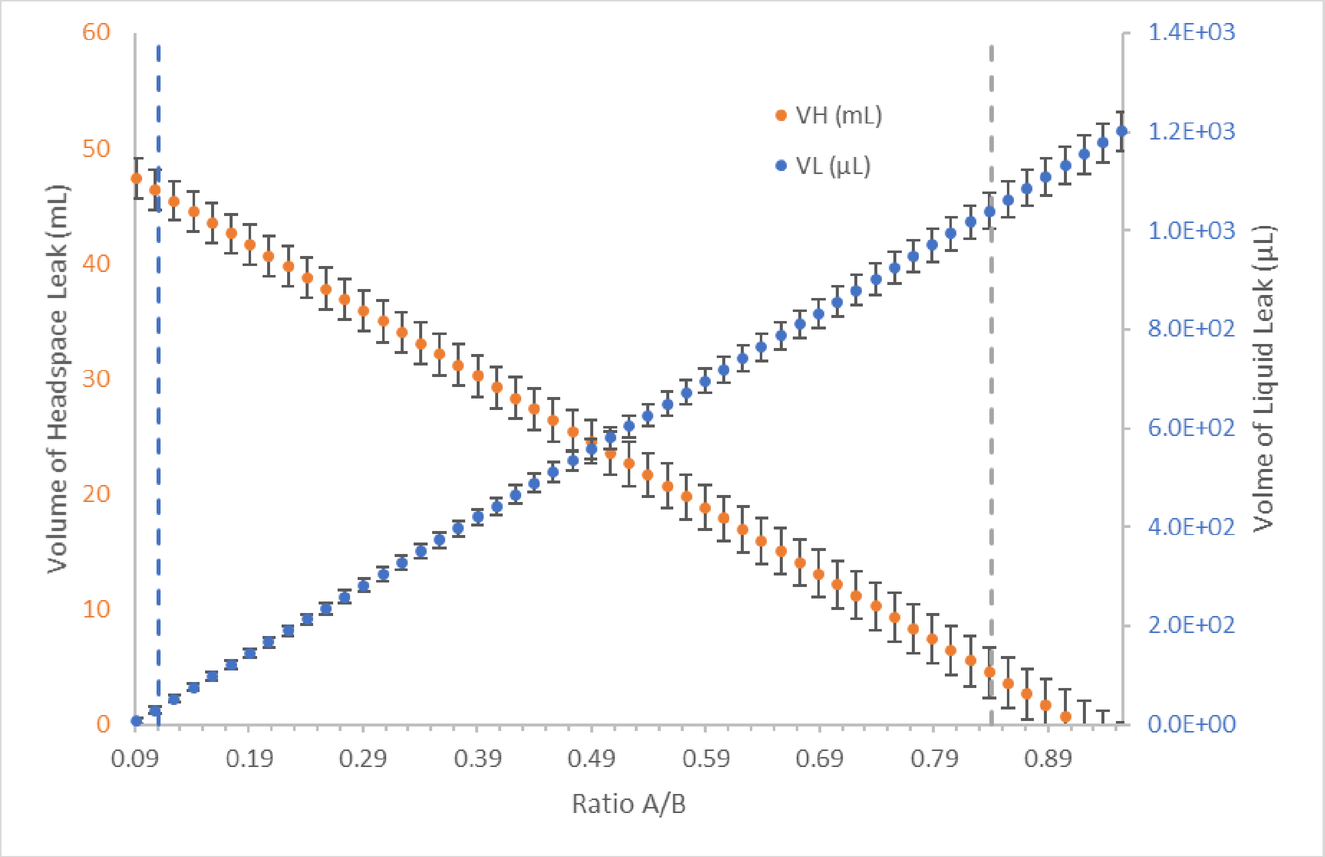

Figure S5. Plot of volume with error bars representing one standard deviation for a large leak with ΔB_T at 100 ppmv and ΔA_T over a range from 90.5 ppmv to 9.5 ppbv to achieve the ratio of A/B over a range from pure headspace leaks to pure liquid leaks. The resulting leak volume of liquid (VL) and headspace (VH) for a given ratio of A/B are shown on the vertical axes.
