# Supplementary material for: Source apportionment and quantification of liquid and headspace leaks from closed system drug-transfer devices via Selected Ion Flow Tube Mass Spectrometry (SIFT-MS)
Source: PLoS One. 2021 Nov 4;16(11):e0258425. doi: 10.1371/journal.pone.0258425 (PMC8568112; doi:10.1371/journal.pone.0258425)
Supplement: S7 Fig — (PDF) [file pone.0258425.s007.pdf]

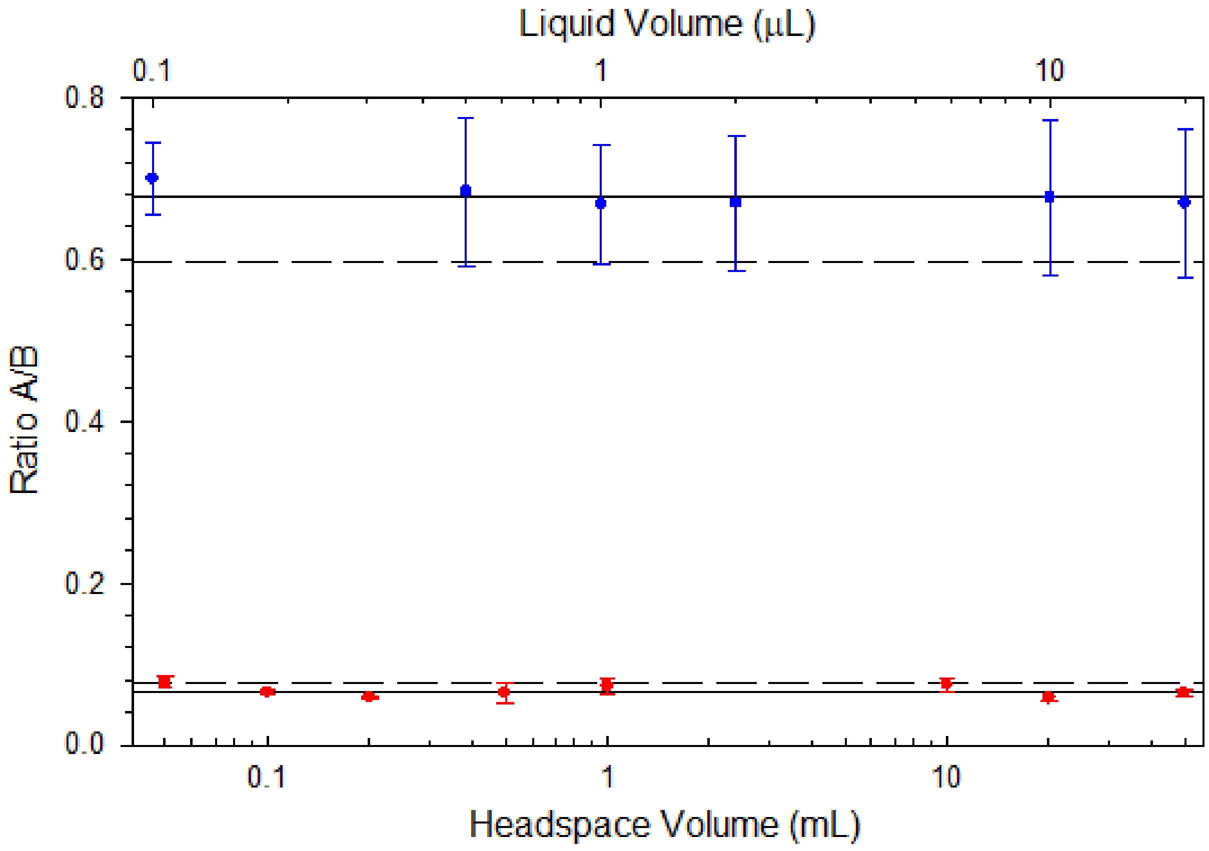

Figure S7. Ratio of mass A / mass B versus volume of liquid (upper line) and headspace (lower line) aliquots.
