# Supplementary material for: Source apportionment and quantification of liquid and headspace leaks from closed system drug-transfer devices via Selected Ion Flow Tube Mass Spectrometry (SIFT-MS)
Source: PLoS One. 2021 Nov 4;16(11):e0258425. doi: 10.1371/journal.pone.0258425 (PMC8568112; doi:10.1371/journal.pone.0258425)
Supplement: S1 Table — (PDF) [file pone.0258425.s008.pdf]

S1 Table. Analysis Method Settings and Configuration of the SIFT-MS.

The SIFT-MS sample inlet was a Syft HPI inlet, which contained a critical orifice which set the inlet flow rate at 20 mL/min. The critical orifice at the sample inlet could not be easily changed while the SIFT-MS was operating. The following table lists settings for the SIFT-MS. The SIFT-MS was operated in a Selected Ion Mode (SIM), scanning only the ions of interest. The library of reagent ions, reaction rate, branching ratios, and product ions used for analysis of the compounds of interest are listed. The product ions that were scanned and measured to calculate a quantitative result are noted in the last two columns. These parameters are typical of the library entries for the LabSyft software (Syft Technologies, Christchurch NZ) used to create methods for the SIFT-MS.

Compound	Reagent Ions	Reaction Rate	Branching ratio (%)	Product Mass (m/z)	Product Ions	Scan	Calculate
1,2-propanediol	H3O+	3.5E-9	95	59	C3H7O+	no	no
1,2-propanediol	H3O+	3.5E-9	5	77	C3H9O2+	yes	no
1,2-propanediol	NO+	2.9E-9	100	75	C3H7O2+	yes	yes
1,2-propanediol	O2+	2.0E-9	85	45	C2H5O+	yes	yes
1,2-propanediol	O2+	2.0E-9	5	59	C3H7O+	yes	yes
1,2-propanediol	O2+	2.0E-9	5	76	C3H8O2+	yes	yes
acetone	H3O+	3.9E-9	100	59	C3H7O+	yes	no
acetone	H3O+	3.9E-9	s.p.	77	(CH3)2CO.H+.H2O	no	no
acetone	NO+	9.0E-10	100	88	NO+.C3H6O	yes	no
acetone	O2+	1.7E-9	65	43	C2H3O+	yes	no
acetone	O2+	1.7E-9	35	58	C3H6O+	yes	yes
methyl tert-butyl ether	H3O+	3.0E-9	70	57	C4H9+	yes	no
methyl tert-butyl ether	H3O+	3.0E-9	30	89	C5H12O.H+	yes	yes
methyl tert-butyl ether	H3O+	3.0E-9	s.p.	107	C5H12O.H2O.H+	no	no
methyl tert-butyl ether	NO+	2.5E-9	84	57	C4H9+	yes	no
methyl tert-butyl ether	NO+	2.5E-9	16	86	C4H8.NO+	yes	no
methyl tert-butyl ether	O2+	2.3E-9	21	57	C4H9+	no	no
methyl tert-butyl ether	O2+	2.3E-9	76	73	C4H9O+	no	no
methyl tert-butyl ether	O2+	2.3E-9	3	89	C5H13O+	no	no

SIFT-MS Tuning Procedure: As concentrations increase, complex secondary reactions can occur. This can obfuscate the relationship between analyte concentration and product ion counts. If the concentration of reaction products from a single reagent ion differ, then the branching ratio was adjusted until all products produced the same concentration. Then the reaction rate (k) for the reagent ion was adjusted, which will affect all product concentrations in the same way, until the correct concentration was obtained. There may be some nonlinearity of the response across a range of concentrations. The severity of the non-linearity may vary among the of product ions. In the work herein, the product ions were chosen that were the most linear across the concentration range of the analytes of interest and adjusted the reaction rates to best fit the results measured by the SIFT-MS to known concentrations of the analytes.
