# Supplementary material for: Source apportionment and quantification of liquid and headspace leaks from closed system drug-transfer devices via Selected Ion Flow Tube Mass Spectrometry (SIFT-MS)
Source: PLoS One. 2021 Nov 4;16(11):e0258425. doi: 10.1371/journal.pone.0258425 (PMC8568112; doi:10.1371/journal.pone.0258425)
Supplement: Attachment — Submitted filename: PLOS ONE Decision.docx [file pone.0258425.s009.docx]

Dear Dr. Doepke,

Thank you for submitting your manuscript to PLOS ONE. After careful consideration, we have decided that your manuscript does not meet our criteria for publication and must therefore be rejected. Please look at the comments made the reviewers to further enhance your publication. Due to the bulk of comments, I suggest to submit it as a new submission and not a revision.

I am sorry that we cannot be more positive on this occasion, but hope that you appreciate the reasons for this decision.

Yours sincerely,

Mannix Balanay, Ph.D.

Academic Editor

PLOS ONE

[Note: HTML markup is below. Please do not edit.] Reviewers' comments:

Reviewer's Responses to Questions

**Comments to the Author**

1. Is the manuscript technically sound, and do the data support the conclusions?

The manuscript must describe a technically sound piece of scientific research with data that supports the conclusions. Experiments must have been conducted rigorously, with appropriate controls, replication, and sample sizes. The conclusions must be drawn appropriately based on the data presented. Reviewer #1: Yes

Reviewer #2: Partly

Reviewer #3: No

2. Has the statistical analysis been performed appropriately and rigorously?

Reviewer #1: Yes

Reviewer #2: Yes

Reviewer #3: Yes

3. Have the authors made all data underlying the findings in their manuscript fully available?

The [PLOS Data policy](http://www.plosone.org/static/policies.action#sharing) requires authors to make all data underlying the findings described in their manuscript fully available without restriction, with rare exception (please refer to the Data Availability Statement in the manuscript PDF file). The data should be provided as part of the manuscript or its supporting information, or deposited to a public repository. For example, in addition to summary statistics, the data points behind means, medians and variance measures should be available. If there are restrictions on publicly sharing data—e.g. participant privacy or use of data from a third party—those must be specified.

Reviewer #1: Yes

Reviewer #2: Yes

Reviewer #3: Yes

4. Is the manuscript presented in an intelligible fashion and written in standard English?

PLOS ONE does not copyedit accepted manuscripts, so the language in submitted articles must be clear, correct, and unambiguous. Any typographical or grammatical errors should be corrected at revision, so please note any specific errors here.

Reviewer #1: Yes

Reviewer #2: No

Reviewer #3: No

5. Review Comments to the Author

Please use the space provided to explain your answers to the questions above. You may also include additional comments for the author, including concerns about dual publication, research ethics, or publication ethics. (Please upload your review as an attachment if it exceeds 20,000 characters)

Reviewer #1: This is a very thorough study showing how SIFT-MS can be used for leak testing of Closed System Drug-Transfer Devices. The results are based on well designed and carefully carried out experiments.

The discussion including aspects such Henrys law and ratios of the two test compounds and observed non-linearity is logical and complete.

The article should be published after the authors had a chance to add the following information to the manuscript:

1) Which reagent and product ions were used for acetone and MTBE? H3O+ is suitable for acetone given m/z 59 and 77 but less so for methyl butyl ether (see Spanel & Smith IJMS 172, 1998, 239-247) where fragment ions are formed. NO+ would presumably be a more suitable regent (m/z 88 for acetone, m/z 108 for MTBE). From the supplementary table it seems that O2+ was used for calculation of acetone from m/z 58 only, this may be a potential source of overlaps with fragments from larger molecules. Anyway, for this study it does not really matter, however the explanation on the last page of Supplemental Materials is not clear to this referee,
2) The relevant references should be cited for the SIFT-MS method, especially quantification of acetone and ethers.
3) Why was not the sample flow rate reduced to avoid issues with large concentrations?

Reviewer #2: The present work aims to present a new method to determine the origin of hazardous drugs leaks (liquid or headspace) from closed system drug-transfer devices (CSTD). The author based the leaks origin detection on the differences of vaporization between two model molecules (acetone and methyl-t-butyl-ether). Both volatile molecules were introduced at the same concentration in water with Propylene Glycol as surrogate in the CSTD. According to Henry’s law, the authors estimated the vapor or liquid leaks origin with SIFTMS measurement in a 131L tight assembly. The ratio Acetone with MTBE give information on leaks origin: same concentration in gas phase means that a liquid leak appears whereas lowest concentration of MTBE than Acetone means a headspace leak during manipulation of the CSTD. This strategy to identify liquid or headspace leaks was already used for inorganic compounds. This work is very interesting but major modifications to be published: Global presentation looks like normative guidelines and should be moved to peer review article with conventional units. There is too much graphs without the appropriate units. For example: Figure 1 should move in SI, figure 2 should represent in horizontal axis the expected concentration of VOC (calculated by the conversion of liquid volume to ppbV) and in vertical axis the measured concentration (ppbV). There are only 5 citations within 3 of NIOSH. Author should cite peer review articles in the field (Wilkinson AS. etal 2018 for example) The calibration of the SIFT-MS was obtained with dilutions of neat acetone and MTBE in tedlar bags with gastight syringes. k values allowing conversion between produced ion count to concentration with SIFT-MS were determine for a wide range of concentrations. This is the first limitation of this instrument which linearity is well known to be correct in a small range of concentration. Authors should determine several k values for several concentration ranges. Is the water concentration considered? This will also affect the calibration. Second limitation: the authors chosen produced ions which have the better linearity on the wide range of concentration (data not shown in SI). Thus, the quantification was not done with the most relevant ions. In a simple air matrix with two molecules, the main produced ions should give more accurate quantification. Third: the differences between expected concentration of injected VOC and measured concentrations is surprising. Since the SIFT calibration seems to be accurate, such a difference could come from the chosen ions for quantification. What is the delay between liquid injections and SIFT-MS measurements. Is the SIFT-MS connected to the assembly during all the experiment? The sampling flow of SIFT-MS is fixed to 20 mL/min that mean that in 1 400 minutes as presented in figure 1, authors collected 28 L of the 131 L total volume. An air compensation should be connected otherwise a pressure drop should occur and the sampling flow will decrease.

The annotations “change in the acetone (_Δ_A) and MTBE (_Δ_B)” are not clear. A simple acetone concentration A will be better. Atotal = AL + AH

Several molecules were cited in this paper and SI and not used. (tetraethylurea, 1,2propanediol). Fluorescein was also used to observe liquid leaks but not developed. It makes confusion.

Authors should describe how they manage the “real test” of CSTD and represent the results

(fig7,8 and9) in bar graph with ratio A/B for each step

To conclude, this represent a large amount of experiment for interesting applications but is confuse. Author should present their result according to peer review papers recommendation and extract the most relevant results.

Reviewer #3: The submitted paper PONE-D-21-05377 describes an important and interesting experimental/data-interpretation work related to the set-up of a method based on SIFT-MS analysis to detect leaks of drugs from closed system drug-transfer devices. The research carried out by the authors is valuable, however the manner in which such research, experiments, and results are presented in the manuscript is in my opinion not adequate to a scientific journal. The research is presented more as an internal laboratory report, than as a

scientific article targeting a wide range of multidisciplinary readers. For this reason my suggestion to the Editor is to reject the manuscript. In order to publish the experiments and the conclusions in the form of a scientific publication, a major revision is not sufficient: the authors need to completely reorganize, rewrite, and integrate the corpus of the manuscript.

This could be a new submission, not a revision.

In particular, the critical issues are in the following aspects, that make the manuscript not easily readable and not understandable by a wide scientific audience of readers. - The introduction does not present any state-of-the-art and does not contextualize the presented experiments in the frame of existing literature

- bibliography is insufficient and extremely partial
- the introduction and the abstract fail in explaining clearly the experimentation. Examples are the followings. The information that SIFT-MS only analyses the VOCs in vapour phase is not included. The fact that the differentiation between liquid and vapour leaks is based on the time evolution changes of the concentration of the VOCs is also not included. Reading the introduction, it is not possible to understand the rationale behind the experiments.
- Abbreviations are not correctly explicated, starting from NIOSH that appears in the first linesof the introduction without any explanation of what it is.
- The description of calibration is quite confusing: lines 73-77 at pag 8 describe a calibration against concentration in Tedlar bags. While at pag 10 calibration against volumes are discussed. What are the first calibrations used for?
- If the change of concentration in time is used to determine if a leak involves a vapour or aliquid, how is the model applicable also when the time at what the leak occurred is not known? - The conclusion are quite generic and general: how is the model extended to other drugs and chemical mixtures?

6. PLOS authors have the option to publish the peer review history of their article ([what does this mean?](https://journals.plos.org/plosone/s/editorial-and-peer-review-process#loc-peer-review-history)). If published, this will include your full peer review and any attached files.

If you choose “no”, your identity will remain anonymous but your review may still be made public.

**Do you want your identity to be public for this peer review?** For information about this choice, including consent withdrawal, please see our [Privacy Policy](https://www.plos.org/privacy-policy).

Reviewer #1: **Yes:** Patrik Španel

Reviewer #2: No

Reviewer #3: No

[NOTE: If reviewer comments were submitted as an attachment file, they will be attached to this email and accessible via the submission site. Please log into your account, locate the manuscript record, and check for the action link "View Attachments". If this link does not appear, there are no attachment files.]

- - - - -

For journal use only: PONEDEC3

*In compliance with data protection regulations, you may request that we remove your personal registration details at any time.* [*(Remove my information/details).*](https://www.editorialmanager.com/pone/login.asp?a=r) *Please contact the publication office if you have any questions.*
