# Supplementary material for: Source apportionment and quantification of liquid and headspace leaks from closed system drug-transfer devices via Selected Ion Flow Tube Mass Spectrometry (SIFT-MS)
Source: PLoS One. 2021 Nov 4;16(11):e0258425. doi: 10.1371/journal.pone.0258425 (PMC8568112; doi:10.1371/journal.pone.0258425)
Supplement: Attachment — Submitted filename: Response to Reviewer.docx [file pone.0258425.s010.docx]

Authors Responses to Reviewer Comments.

Reviewer's Responses to Questions

**Comments to the Author**

1. Is the manuscript technically sound, and do the data support the conclusions?

The manuscript must describe a technically sound piece of scientific research with data that supports the conclusions. Experiments must have been conducted rigorously, with appropriate controls, replication, and sample sizes. The conclusions must be drawn appropriately based on the data presented.

Reviewer #1: Yes

Reviewer #2: Yes

Reviewer #3: Yes

2. Has the statistical analysis been performed appropriately and rigorously?

Reviewer #1: Yes

Reviewer #2: Yes

Reviewer #3: Yes

3. Have the authors made all data underlying the findings in their manuscript fully available?

The [PLOS Data policy](http://www.plosone.org/static/policies.action#sharing) requires authors to make all data underlying the findings described in their manuscript fully available without restriction, with rare exception (please refer to the Data Availability Statement in the manuscript PDF file). The data should be provided as part of the manuscript or its supporting information, or deposited to a public repository. For example, in addition to summary statistics, the data points behind means, medians and variance measures should be available. If there are restrictions on publicly sharing data—e.g. participant privacy or use of data from a third party—those must be specified.

Reviewer #1: Yes

Reviewer #2: Yes

Reviewer #3: Yes

4. Is the manuscript presented in an intelligible fashion and written in standard English?

PLOS ONE does not copyedit accepted manuscripts, so the language in submitted articles must be clear, correct, and unambiguous. Any typographical or grammatical errors should be corrected at revision, so please note any specific errors here.

Reviewer #1: Yes

Reviewer #2: No

Reviewer #3: No

The reviewers did not mention any specific errors.

5. Review Comments to the Author

Please use the space provided to explain your answers to the questions above. You may also include additional comments for the author, including concerns about dual publication, research ethics, or publication ethics. (Please upload your review as an attachment if it exceeds 20,000 characters)

Reviewer #1: This is a very thorough study showing how SIFT-MS can be used for leak testing of Closed System Drug-Transfer Devices. The results are based on well designed and carefully carried out experiments. The discussion including aspects such Henrys law and ratios of the two test compounds and observed non-linearity is logical and complete.

The authors revised the manuscript according to the reviewer comments on the original submission.

We did our best. Thank you.

The explanation on the last page of the Supplemental Materials is more clear now.

It should be mentioned that for this application, reduced sample flow rate would result in better linearity. There is a comment "Commented [DA(3]: Our instrument has a fixed (nonadjustable) sample inlet flow rate. I will mention this in the manuscript." but it is not really discussed. The actual flow rate used should be stated.

The flow rate used was previously listed in the methods section, additionally comments have been added to the discussion related to increasing linearity by adjusting the sample inlet flowrate.

Reviewer #2: This manuscript appears to describe a technical method for leak testing of closed system drug-transfer devices using SIFT-MS. The manuscript is a resubmission (following initial rejection), and the previous reviewers' comments are appended, with the author's comments and thoughts included as track changes.

While some of the reviewers comments seem to have been addressed, as reviewer 3 stated "The research is presented more as an internal laboratory report, than as a scientific article targeting a wide range of multidisciplinary readers.". This does not appear to have been addressed in any meaningful way.

References were added to detail where we fit into the available literature that may be of interest to multidisciplinary readers. The discussion and conclusion have been revised to address this. In the conclusion we have reiterated how this research may be impactful to multidisciplinary readers within the CSTD community.

This seems to be a stylistic critique. We are trying to present a technical paper since the focus of the research is on the method. Reviewer three wants commentary on CSTD testing, and an in-depth research report, and comparison with literature. We can only do so much without expanding the intended scope of the article.

Reviewer #3: The authors were well replied to technical comments and the paper was corrected as suggested by the referees. However some specific remarks are mandatory to enhance the quality of this manuscript.

The paper would be easier to understand if the details of each figure and table are explained and discussed (they should be more explained and detailed). In general, the authors provide a somewhat clear description of what was done.

It is not clear from the reviewer’s comment which details are lacking, though we have added more detail in the captions of the figure and tables.

Experimental results are presented in a vague manner. Some justifications and more information about the calibration and quantification results should be given, otherwise this is a serious problem because there is no way to understand how those results are robust and accurate.

We are having some uncertainty to which “results” the reviewer is referring to. We have commented on two possibilities; results of the calibrations and the analysis of the instrument response, or the results from testing CSTDs.

The experimental results involving CSTDs was not meant to be a complete evaluation of any of the devices but was presented as examples of the data that could be obtained using this method. Several replicates of CSTD testing would have been required to provide a picture of the robustness of the results from CSTD testing, which is a topic for another publication. A set of calibrations was performed daily to characterize the instrument response before conducting the experiments involving CSTDs (which has been added to the paper).

From the detailed analysis of aggregated calibration data, we characterized the instrumental uncertainties of the SIFT-MS. The aggregated calibration data from replicates of calibration procedures and the presentation of the variability encountered with this method was the focus of this publication. We tried to very explicitly spell out the uncertainties associated with this method. The calibration results are aggregated replicates of six. While the discussion does not comment on each of the data points individually, Table 1. Provides relevant metrics of the performance of the calibration which are the indicators of accuracy and robustness. Additional details are provided in the section “*Ratio of A/B from slopes of the calibration curves*” and in the section “*Average ratio of A/B from all calibration measurements.”.* Further in the discussion, uncertainty analysis based upon the calibration data is discussed in detail along with theoretical examples of the uncertainties related to various leak scenarios that might be encountered.

The discussion is incomplete or even missing, does not clearly present the authors' conclusions, and make some broad generalizations that are not based upon the data presented. It should be re-written to provide a much clearer interpretation of results comparing them with previous findings in this filed. Relevant references should be included and discussed.

It is unclear which statements the reviewer is referring to when saying, “broad generalizations that are not based upon the data presented”.

In the previous revision of this paper in response to the reviewer request, we did add relevant references and discuss how this research fits in with the available literature in the introduction. It is somewhat beyond to the scope of this paper to go into a detailed interpretation of others work. In general, we feel it is best to limit the scope to interpretation of the results presented in this work.

We have revised both the discussion and conclusion sections to address the reviewer’s comments.

In addition, in the conclusion section, a critical evaluation of the advantages and limitations of the SIFT-MS technique,

The advantages and limitations of the SIFT-MS as it pertains to this application should be paramount in our publication and we probably felt it already was included in the detailed investigation of the SIFT-MS calibration response data.

Although in light of the reviewer comments, we have added to the publication that, the SIFT-MS has low (ppt-ppb) limits of detection for VOCs and semi-volatiles, the issue we encountered was one of consistent transfer of semi-volatiles to the instrument. The semi-volatiles with similar volatility as HDs seemed to deposit on the surfaces of the experimental setup rather than being quantitatively transferred to the SIFT-MS, which precluded us from achieving our intended goal of measuring a compound with similar volatility as the actual HDs.

A more general assessment of the SIFT-MS technique would be beyond the scope of the paper.

the future perspectives and the expected outcomes in the field of leak testing of Closed System Drug-Transfer Devices are necessary and strongly recommended.

I have to remind the reviewers that the research presented are the opinions of the authors and not necessarily representative of NIOSH policy. Future techniques might have the additional power to discriminate between liquid leaks and vapor leaks as well as assessing the containment via vapor from air filtering CSTDs. We concede that we have not proven or disproven the containment efficacy of any of filter based CSTDs, we do see the need for complimentary techniques to assess filtration devices. While not a definitive end point for CSTD testing this research provides a framework for future research which may come closer to providing a complete picture of the efficacy of any type of CSTDs.

6. PLOS authors have the option to publish the peer review history of their article ([what does this mean?](https://journals.plos.org/plosone/s/editorial-and-peer-review-process#loc-peer-review-history)). If published, this will include your full peer review and any attached files.


If you choose “no”, your identity will remain anonymous but your review may still be made public.


**Do you want your identity to be public for this peer review?** For information about this choice, including consent withdrawal, please see our [Privacy Policy](https://www.plos.org/privacy-policy).

Reviewer #1: **Yes:** Patrik Španěl

Reviewer #2: No

Reviewer #3: No
